# Survival and prognosis of neurofibromatosis type 1-associated malignant peripheral nerve sheath tumours: a systematic review and meta-analysis

**DOI:** 10.1186/s13023-026-04460-w

**Published:** 2026-06-17

**Authors:** Mette Møller Handrup, Ninna Aggerholm-Pedersen, Stine Bogetofte Thomasen, Emma Hyldgaard Olesen, Cecilie Ejerskov

**Affiliations:** 1 Center for Rare Diseases, Department of Pediatric and Adolescent Medicine, Aarhus University Hospital, Aarhus, Denmark; 2 Department of Oncology, Aarhus University Hospital, Aarhus, Denmark; 3 Department of Clinical Medicine, Aarhus University, Aarhus, Denmark

**Keywords:** Neurofibromatosis type 1, Neurofibrosarcoma, Survival rate, Prognosis

## Abstract

**Background:**

Malignant peripheral nerve sheath tumour (MPNST) is a rare, aggressive sarcoma with high mortality. MPNST can develop sporadically, after radiation therapy or in association with neurofibromatosis type 1 (NF1). The treatment is challenging especially if surgical removal is not possible. NF1 is an autosomal-dominant genetic disorder most often caused by a germline pathogenic variant in the *NF1* gene and in rare cases a deletion of the *NF1* gene. Patients with NF1 have a higher risk of developing several different cancers, of which MPNST is one of the most frequent. MPNST in individuals with NF1 often presents with large MPNST which are often not accessible for surgery. Several studies have shown that patients with NF1-associated MPNST (nfMPNST) have an overall inferior survival than those with sporadic MPNST (sMPNST). Despite this, NF1 status alone may not be a causative factor for poor prognosis, which might rather be due to the incidence of larger tumours, which are more challenging to resect in toto.

**Methods and results:**

A systematic research protocol was made using the PRISMA-P model and the review question and inclusion and exclusion criteria were defined using the PICO model. The study characteristics defined by PICO include patients with NF1 as the population of interest. The development of MPNST was considered as the intervention, patients with sMPNST were chosen for comparison and the primary outcome of interest was survival. The literature search was performed on 12 October 2024 and 4,394 studies were eligible for screening, of which 36 studies were included in this study. Meta-analysis of eight studies found NF1 status to be a risk factor for the survival of MPNST. The reported survival rates varied between studies, but the 5-year overall survival (OS) remained poor in general, Awith 15 studies showing a significantly inferior survival for nfMPNST. Only four studies have a worse survival for sMPNST, but none with a significant difference.

**Conclusion:**

The findings in this systematic review and meta-analysis of 36 studies indicates that patients with nfMPNST have a worse survival compared to sMPNST.

**Supplementary Information:**

The online version contains supplementary material available at https://doi.org/10.1186/s13023-026-04460-w.

## Introduction

Malignant peripheral nerve sheath tumour (MPNST) is a rare, aggressive sarcoma which accounts for 5–10% of all soft tissue sarcomas [1, 2]. They can arise anywhere in the body but are commonly located in the extremities, the trunk and the head and neck, deeply seated in the soft tissue near a nerve [3]. The prognosis is generally poor, with high mortality, and the tumours have a high tendency of local recurrence and metastasis [4–6]. MPNST can develop sporadically, after radiation therapy or in association with neurofibromatosis type 1 (NF1), which accounts for up to 50% of the cases [7]. The treatment of MPNST is challenging among all age groups due to the low efficacy of conventional systemic chemo- and radiation therapy and the high tendency of metastasis. The most effective therapy for MPNST is surgical resection with wide margins which is often not feasible due to location or size, the potential associated morbidity of surgery, or the presence of distant metastases [8].

NF1 is an autosomal-dominant genetic disorder most often caused by a germline pathogenic variant in the *NF1* gene and in rare cases a deletion of the *NF1* gene, the latter with a particularly increased risk of cancer [9]. With an incidence of one case per 2,500 to 3,000 individuals worldwide, NF1 is relatively common among rare diseases. It has a wide range of clinical manifestations, and the diagnostic features often develop during childhood [10, 11].

Patients with NF1 have a higher risk of developing several different cancers such as breast cancer, gliomas and MPNST, the latter being one of the most frequent. Patients with NF1 have a lifetime risk of developing MPNST of 8–13% compared to 0.001% in the general population [12], and they develop MPNST at a significantly younger age [4, 5]. MPNST can arise from a spontaneous mutation in peripheral nerve sheaths or a malignant transformation of a pre-existing benign tumour, such as plexiform neurofibromas, which are present in 30–50% of patients with NF1 [13–15]. MPNST in NF1 patients can be challenging to diagnose because clinical indicators of malignancy, such as pain, a growing mass, and neurologic compromise, may also be features of pre-existing plexiform neurofibromas [5].

Several studies have shown that patients with nfMPNST have an overall inferior survival than those with sMPNST. NF1-associated MPNST typically develops through a sequence of premalignant stages (plexiform neurofibroma → atypical neurofibroma → ANNUBP (atypical neurofibromatous neoplasm of uncertain biologic potential) → MPNST), driven by multiple genetic hits. This stepwise progression makes the disease more complex and likely more difficult to recognise clinically, which may explain the poorer outcomes observed in nfMPNST [16–18]. Despite this, NF1 status alone may not be a causative factor for poor prognosis which might rather be due to the incidence of larger tumours, which are more challenging to resect in toto [19]. The role of NF1 status and prognosis is therefore still debated, and it is yet to be resolved whether nfMPNST has an inferior outcome and should be managed differently from sMPNST.

This systematic review and meta-analysis summarize the current knowledge on survival for nfMPNST compared to sMPNST and assess the prognostic role of NF1 status.

## Methods

### Search strategy

A systematic research protocol was made using the PRISMA-P checklist to answer the research question and identify all eligible studies [20]. The review question and inclusion and exclusion criteria were defined using the PICO model [21]. The study characteristics defined by PICO include patients with NF1 as the population of interest. The development of MPNST was considered as the intervention. For comparison, patients with sMPNST were chosen and the primary outcome of interest was survival. Based on these, a systematic literature search was conducted using the databases PubMed and EMBASE (see supplementary I for details). The search string included MeSH terms and synonyms related to NF1 and MPNST.

Study designs to be included were cohort studies, case/control studies, case reports, and randomised controlled trials. The inclusion criteria were: (1) studies with original data, (2) studies with at least one measurement of survival and (3) studies with clinicopathological information on: gender, age, follow-up period and NF1 status. The exclusion criteria were: (1) reviews, meta-analyses, conference abstracts, guidelines, letters, comments, or editorials without original data, (2) studies with less than 10 patients with either subgroup nfMPNST or sMPNST, (3) animal studies or in vitro studies and (4) studies without measurements of survival for each of the two patient groups.

Limits applied to the search were as follows: age: no limit; species: only human; gender: both; time frame: from 1990 to 2024; language: English. An asterisk (*) was applied to each search string to incorporate both spellings of tumor/tumour.

In order to be sure to include all relevant literature in the search, no terms related to “survival” were added to the search strings, since many studies include measurements of survival in their data analysis without it being their main aim of the study.

### Selection process

All records were managed through the online systematic review software Covidence. Studies were first screened for eligibility by title and abstract. A total of 20 studies were randomly selected and screened independently by five researchers using the inclusion and exclusion criteria and any disagreements were resolved in consensus. Two researchers independently screened the remaining studies. Any disagreements were resolved in consensus by these two. The same process was repeated for studies eligible for full-text reading.

### Data extraction

The primary extracted outcomes were measurements of survival calculated as a 5-year OS percentage and/or univariate cox proportional hazard ratio (HR) for each of the subgroups: nfMPNST and sMPNST. Studies describing survival as disease-specific survival (DSS) were pooled separately.

For studies without measurements of OS, but with information on the follow-up period and status at end of follow-up (alive/dead), data was extracted to STATA and a 5-year OS and HR with CI 95% intervals were calculated using the survivor function and Cox proportional hazard model.

### Quality assessment

The quality of evidence for each eligible study was assessed individually by two researchers using the Newcastle-Ottawa Scale (NOS), which evaluates studies across three domains: Selection, Comparability, and Outcome/Exposure. Each study was assigned stars according to the NOS manual [22].

### Statistical analysis

Analyses were performed using Stata 17.0. Different measurements of survival were grouped and presented as ranges. Cochran’s *Q* test was used to assess heterogeneity between studies and the result was quantified using an *I*^*2*^ statistic. A value of *I*^2^ < 50% and *P* > 0.1 would indicate that no statistical significance of heterogeneity was present. A random effect model was used to pool univariate hazard ratio (HR) values of NF1 status as a prognostic factor. If HR > 1, then NF1 was accepted as a risk factor resulting in poor prognosis. Egger’s test was used to assess the presence of publication bias. A value of *P* > 0.05 was accepted as no statistically significant publication bias.

## Results

**Fig. 1:**
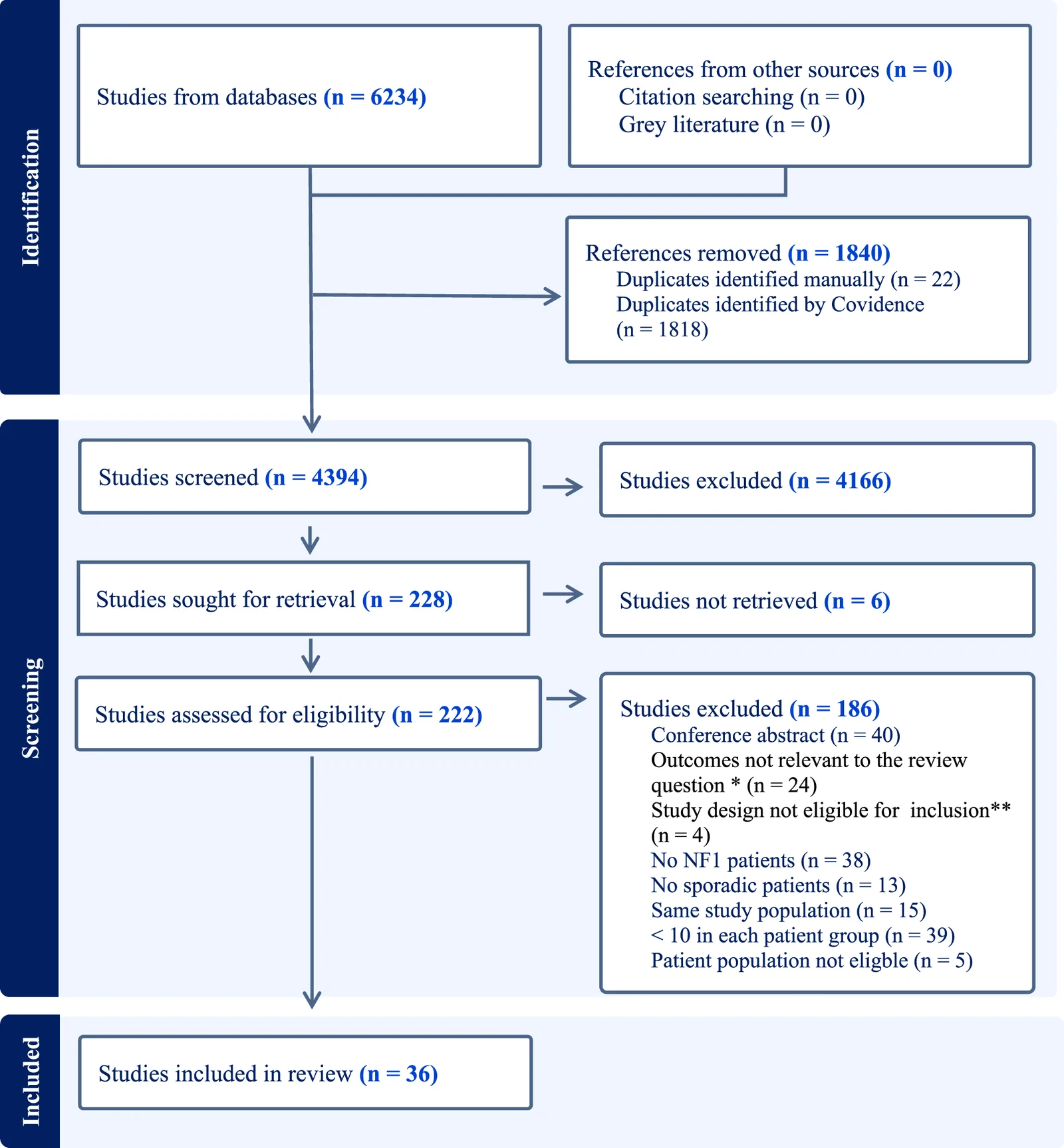
Flow chart showing the selection process. *Studies without measurements of survival or prognosis. **Reviews, meta-analyses, conference abstracts, guidelines, letters, comments or editorials without original data and case reports with less than 10 patients

The literature search was performed on 12 October 2024 and 4,394 studies were eligible for screening (Fig. 1). After screening by title and abstract, 228 studies were sought for retrieval. Six studies were not possible to access for full-text screening and were excluded. After full-text screening, 186 studies did not conform to the inclusion criteria and were excluded. For studies with the same study population, an overlap was assessed when study populations were derived from the same source in overlapping time periods. The study with the biggest and best-represented study population was included. A total of 36 literatures were included in the study.

Characteristics of the included studies are presented in Table 1. Publication years ranged from 1990 to 2023 and covered a time frame from 1945 to 2023. Fifteen different countries were represented, with a total of 4,077 patients.

**Table 1:** Characteristics of included studies

Study	Year	Time frame	Country	Total patients	NF1-MPNST	Sporadic MPNST	Age*†	Quality score	Outcome‡
Agaram, N.P. et al.	2023	2000–2022	USA	64	37	27	< 20	5	OS
Anghileri, M et al.	2006	1976–2003	Italy	205	46	159	37	8	DSS/HR
Brekke, H. R. et al.	2009	1980–2002	Sweden Norway	64	27	35	24/54*	7	OS
Carli, M. et al.	2005	1975–1998	Italy Germany	167	29	138	< 18	8	OS‡
Chang, Yu-Wei et al.	2023	2005–2021	Taiwan	41	12	29	44†	4	OS
Cunha, K. S. G. et al.	2012	1996–2005	Brazil	28	15	13	19–85	7	OS‡/HR
DeCou, J. M et al.	1995	1964–1993	USA	28	11	17	< 29	7	OS/HR
Doorn, P. F. et al.	1995	1977-	Netherlands	22	11	11	32.5	8	OS
Endo, M. et al.	2011	1964–2008	Japan	85	33	52	47	8	OS
Evans, D. G. et al.	2002	1984–1996	England	61	24	37	26/62*	7	OS‡
Fan, Q. et al.	2014	NA	China	146	17	129	NA	6	HR‡
Hagel, C. et al.	2007	1991–2003	Germany	52	38	14	26.5/60.6*	7	OS‡
Holtkamp, N. et al.	2007	NA	Germany	36	22	14	32.6/52.4*	5	OS/HR‡
Hruban, R. H. et al.	1990	1945–1988	USA	43	23	20	40	6	OS
Hwang, I. K. et al.	2017	1988–2015	Korea	95	33	62	40.4†	8	OS/HR
Ikuta, K. et al.	2014	1986–2011	Japan	30	16	14	44.5	8	OS‡
Kahn, J. et al.	2012	1990–2012	USA	33	18	15	25	7	OS
Kamran, S. C. et al.	2013	1999–2011	USA	84	24	60	43.2	8	OS
Kourea, H. P. et al.	1998	1960–1995	USA	25	15	10	31.6	7	DSS
Krawczyk, M. A. et al.	2019	1992–2013	Poland	26	10	16	10.5†	7	OS‡/HR‡
Martin, E. et al.	2020	1989–2017	Netherlands	594	153	441	49	5	OS/HR‡
McCaughan, J. A. et al.	2007	1993–2002	Scotland	25	12	12	42.1/59.0*	5	OS‡
Meis, J. M. et al.	1992	1965–1985	USA	57	13	44	10	5	OS‡
Meister, M. T. et al.	2020	1981–2015	Germany	159	38	121	12.7	8	OS‡
Miao, R. et al.	2019	1960–2016	USA	259	77	182	41	8	OS‡/HR‡
Okada, K. et al.	2007	1994–2002	Japan	56	25	31	45†	8	OS
Porter, D. E. et al.	2009	1979–2002	Scotland England	123	33	90	26/53*	6	OS‡/HR‡
Sharma, M. R. et al.	2021	2011–2018	India	92	12	80	41.5†	7	OS‡
Sobczuk, P. et al.	2020	1998–2018	Poland	239	28	211	51	6	HR
Stucky, C. H. et al.	2012	1985–2010	USA	175	57	114	44	7	DSS/HR
Valentin, T. et al.	2016	1990–2013	France	340	131	209	42	8	OS‡/HR
van Noesel, M. M. et al.	2019	2005–2016	5 European countries	51	26	25	13.7	7	OS‡/HR‡
Vasconcelos, R. A. T. et al.	2017	1990–2010	Brazil	92	41	51	43.5	6	OS‡/HR‡
Wakeman, K. M. et al.	2022	2000–2018	USA	90	36	54	45	6	HR
Watson, K. L. et al.	2017	1990–2014	USA	263	148	115	33/43*	7	HR‡
Zhang, L. et al.	2023	2010–2021	USA	65	41	24	49	3	OS/HR

*NA* not available, *OS* overall survival, *DSS* disease-specific survival, *HR* hazard ratio

* median age for NF1 patients and sporadic patients, respectively

† median age at MPNST diagnosis

‡ significant difference (*P* < 0.05) between the two groups in survival analysis

Twenty-one studies [19, 23–39] presented data on 5-year OS rates for both subgroups. The 5-year OS rate ranged from 0 to 67% for the NF1 group and 37.5–100% for the sporadic group. Seven studies [40–46] presented data as median OS in months, which ranged from 6 to 51 months for the NF1 group and 13–101 months for the sporadic group. Three studies [19, 47, 48] used DSS to measure survival, and the 5-year DSS rates ranged from 20 to 54% for the NF1 group and 12–75% for the sporadic group. One study [49] only presented a 2-year OS rate of 25% for the NF1 group and 68% for the sporadic group (*P* = 0.03). Seventeen [22–24, 26–30, 35, 39, 41, 45], 48– [25] out of 32 studies with survival rates showed a significantly worse OS for nfMPNST compared to sMPNST.

**Fig. 2:**
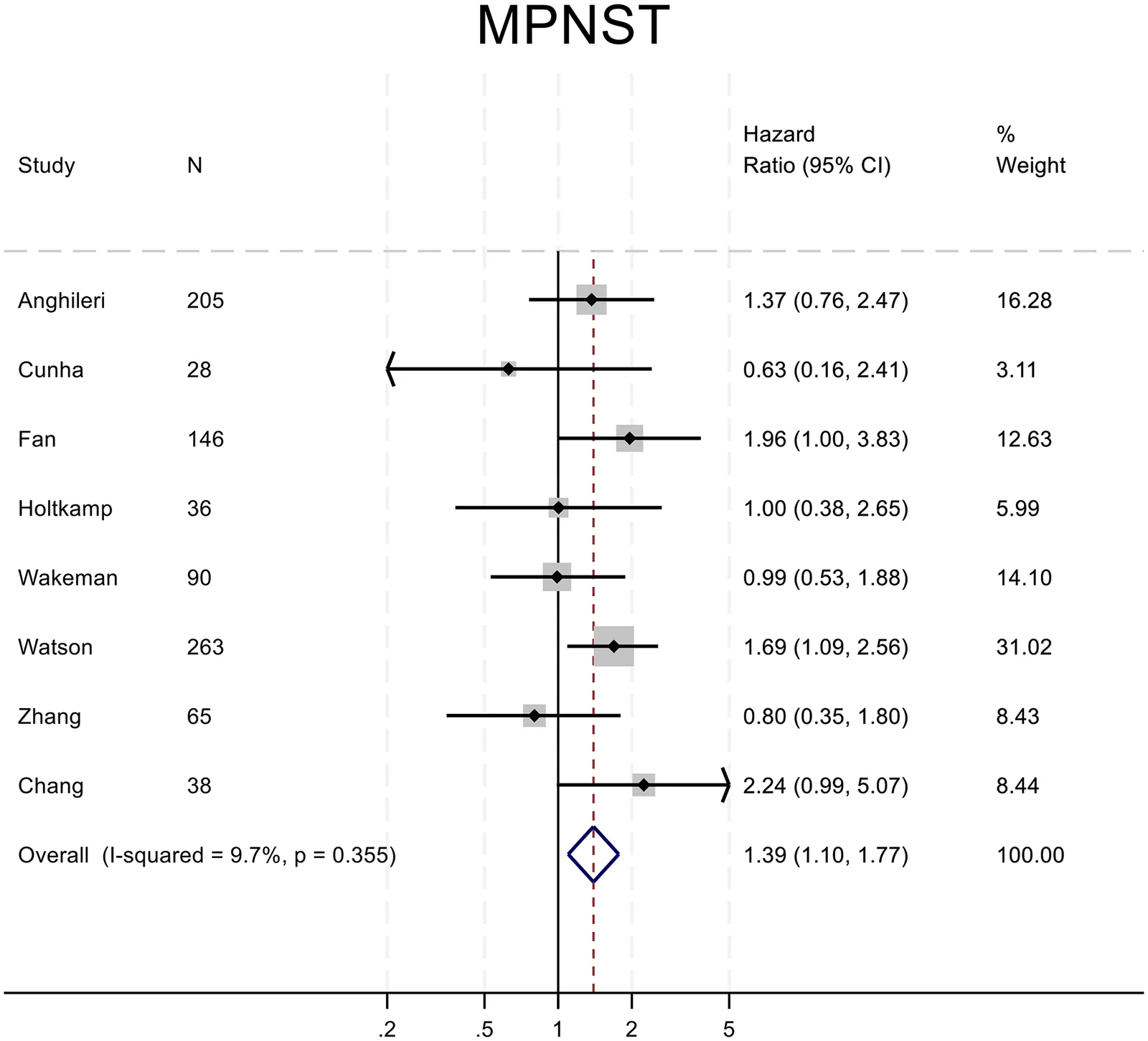
Meta-analysis of pooled HR for NF1 status

Seventeen studies had estimates of the prognostic value of NF1 status [19, 26, 29, 35, 37–40, 45, 48, 50–57]. Nine of them found NF1 to be a prognostic factor of poor prognosis [29, 35, 38, 39, 45, 50–52, 55]. Six studies only presented HR from multivariate analyses and were therefore not included in the meta-analysis [35, 37, 39, 45, 48]. Another five studies were not included in the meta-analysis due to only having paediatric cases [26, 38, 50] or cases of only localized and curative intended disease [37, 53]. A total of eight studies [19, 29, 40, 51, 54, 55] with univariate HR assessing the association with NF1 and OS were pooled in the meta-analysis (Fig. 2). To evaluate the consistency of findings across studies, statical heterogeneity was assessed using the I^2^ statistic and Cochran’s Q test. The I^2^ value of 9.7% indicating low heterogeneity, and the associated p-value was 0.355, suggesting that the observed variation among study results is not statistically significant. These findings imply that the difference in effect estimates across studies are likely attributable to random variation rather than systematic differences in study design, populations and intervention. The pooled result indicates that patients with nfMPNST have a statistically significant poorer survival compared to sMPNST with HR 1.39 (95% CI 1.10–1.77). Egger’s test resulted in *P* = 0.182, indicating that the possibility of publication bias could be excluded.

## Discussion

The prognostic role of NF1 is still highly debated. A meta-analysis by Kolberg M. et al. [16] from 2013 indicates that NF1 status has no effect on survival. However, results from more recent meta-analyses by Cai, Z. et al. from 2020 and by Lim et al. from 2024 indicated that patients with nfMPNST have poorer survival than patients with non-NfMPNST [58, 59]. The present meta-analysis study confirms NF1 as a factor of poor prognosis.

This study summarises the survival data of 4,077 patients from 36 studies with measurements of OS for nfMPNST compared to sMPNST. Meta-analysis of eight studies found NF1 status to be a risk factor for the survival of MPNST, HR 1.39 (95% CI 1.10–1.77). The meta-analysis in this study is based on fewer studies than previous meta-analyses of 28 and 59 studies respectively, due to the inclusion criteria of a minimum of 10 cases in each group [58, 59]. The reported survival rates varied between studies, but the 5-year OS remained poor in general, with 15 studies showing a significantly inferior survival for nfMPNST [22–24, 26–30, 35, 39, 41, 45], 48– [25]. Only four studies had a worse survival for sMPNST, but none with a significant difference [26, 34, 41, 60].

A review from 2020 of the molecular mechanisms underlying MPNST progression highlighting the role of secondary genetic alteration (e.g. *TP53*,* EGFR*) in driving malignant transformation from benign neurofibromas [61]. This might suggest a potential for tumor progression or recurrence following radiation therapy in peripheral nerve sheath tumours. Roohani et al. [62] conducted a retrospective cohort study demonstrating that patients with MPNSTs who presented with local recurrence had significantly worse local control outcomes compared to those with primary localized disease. Yet, for primary MPNST there was a significant benefit of adding radiation therapy to surgery in terms of local control. The addition of radiation therapy to surgery regarding wither or not it gives a better OS is still debatable with contradicting results [63–65]. This could be due to the potential genetic predisposition in MPNST, therefore, more studies on the role of radiation therapy treatment in genetic predisposed patients are needed.

The findings from this systematic review and meta-analysis of 36 studies suggest that patients with nfMPNST experience poorer survival compared with patients with sMPNST. Most included studies reported significantly worse OS among individuals with NF1, and the pooled estimates from the meta-analysis support the hypothesis that NF1 status may represent an adverse prognostic factor in MPNST. Several factors may contribute to this observation. Clinically, nfMPNST frequently arise from pre-existing plexiform neurofibromas and often occur in individuals with a substantial neurofibromatosis tumor burden, which may complicate early detection and management. In addition, symptoms suggestive of malignant transformation may overlap with those of benign neurofibromas, potentially delaying recognition and diagnosis. The underlying genetic predisposition and complex disease phenotype in NF1 may also influence tumor biology and therapeutic outcomes, which together may contribute to more advanced disease at presentation and poorer survival outcomes.

This study included studies with a minimum of 10 cases in each group. This was done to include as many studies as possible since both NF1 and MPNST are relatively rare diseases, resulting in a limited number of reported cases. However, a small study population size can affect the outcomes of a study since the limited number of cases within each study may affect the statistical power and generalisability of the findings. Only six studies of the included studies had a population below 30 cases, and no cases overlapped with other studies. Together with the total number of patients and the broad number of countries in the present study, the inclusion of studies with small study populations is not considered to significantly impact the interpretations of the systematic review results. Several studies were excluded to avoid duplicated study populations, further strengthening the present findings’ reliability.

In the quality assessment, the adequate follow-up period for all included patients was set to be at least two years. This was due to most recurrence events happening within two years after MPNST diagnosis [66]. Several studies did not meet this, and a study might have ended before all possible events had happened, affecting the survival outcome.

There is a risk, particularly with the earlier studies, that some individuals classified as having sMPNST were in fact undiagnosed cases of nfMPNST due to limited access to genetic testing at the time. Improvements in next-generation sequencing technologies, may have enabled earlier and more definitive diagnoses, potentially influencing the ascertainment and clinical characteristics of nfMPNST patients included in this cohort [67]. Furthermore, the diagnostic criteria were revised in 2021 to include the identification of pathogenic variants and/or deletions in the *NF1*-gene. This may also have contributed to a faster diagnosis of individuals with nfMPNST in subsequent years [68].

## Conclusion

The findings in this systematic review and meta-analysis of 36 studies indicate that patients with nfMPNST have a worse survival compared to sMPNST. The majority of the included studies showed significantly worse OS for NF1 patients, and results from the meta-analysis may indicate that NF1 status is a prognostic factor for poor prognosis in MPNST. These observations highlight the clinical importance of vigilant surveillance and prompt evaluation of suspicious or rapidly changing lesions in individuals with NF1. Furthermore, continued efforts to establish large, well-characterized NF1 cohorts and collaborative international datasets will be important to better understand the biological and clinical determinants of prognosis in nfMPNST. Such efforts may ultimately support the development of tailored surveillance strategies and more effective therapeutic approaches for patients with NF1.

Nevertheless, further research is necessary to clarify the prognostic role of NF1 and to identify underlying mechanisms and factors which affect the worse outcome.

## Supplementary Information

Below is the link to the electronic supplementary material.


Supplementary Material 1


## Data Availability

The datasets used and/or analysed during the current study are available from the corresponding author on reasonable request.

## Abbreviations

CI: Confidence interval
DSS: Disease-specific survival
HR: Hazard ratio
MPNST: Malignant peripheral nervous sheath tumour
NF1: Neurofibromatosis type 1
nfMPNST: Neurofibromatosis type 1-associated Malignant peripheral nervous sheath tumour
OS: Overall survival
PICO: Patints/population, intervention, comparison and outcomes
PRISMA-P: Preferred reporting items for systematic review and meta-analysis protocols
sMPNST: sporadic Malignant peripheral nervous sheath tumour
