# Supplementary material for: Survival and prognosis of neurofibromatosis type 1-associated malignant peripheral nerve sheath tumours: a systematic review and meta-analysis
Source: Orphanet J Rare Dis. 2026 Jun 17;21:307. doi: 10.1186/s13023-026-04460-w (PMC13528161; doi:10.1186/s13023-026-04460-w)
Supplement: Supplementary file 1 [file 13023_2026_4460_MOESM1_ESM.docx]

Survival and prognosis of neurofibromatosis type 1-associated malignant peripheral nerve sheath tumours: a systematic review and meta-analysis

**Supplementary I**

A systematic literature search was conducted using the databases PubMed and EMBASE. The search string included MeSH terms and synonyms related to neurofibromatosis type 1 and malignant peripheral nerve sheath tumour and was for each database as follows:

PubMed:

"Neurofibromatosis 1"[Mesh]) OR (neurofibromatosis type I)) OR (neurofibromatosis 1)) OR ("Genes, Neurofibromatosis 1"[Mesh])) OR (neurofibromatoses)) OR (recklinghausen)) OR (NF1)) OR (neurofibromatosis type 1)) AND ("Neurofibrosarcoma"[Mesh])) OR (MPNST)) OR (malignant peripheral nerve sheath tumo*)) OR (neurogenic sarcoma).

EMBASE:

'neurofibromatosis type 1'/exp OR 'neurofibromatosis type i' OR 'neurofibromatosis 1' OR 'neurofibromatoses' OR 'recklinghausen' OR 'nf1' OR 'neurofibromatosis type 1' AND 'neurofibrosarcoma'/exp OR 'mpnst' OR 'malignant peripheral nerve sheath tumo*'.
